# Perioral dermatitis secondary to topical clascoterone 1%

**DOI:** 10.1016/j.jdcr.2025.11.036

**Published:** 2025-12-17

**Authors:** Megan Noda, Munther Zureigat

**Affiliations:** a The Wollongong Hospital, Illawarra Shoalhaven Local Health District, Wollongong, New South Wales, Australia; b Department of Dermatology, Royal Prince Alfred Hospital, Sydney, New South Wales, Australia; c Flinders Street Dermatology, Wollongong, New South Wales, Australia

**Keywords:** acne vulgaris, clascoteroneperioral, dermatitis, drug-induced dermatitis, drug related side effects, topical androgen antagonist

## Introduction

Clascoterone 1% topical cream (Winlevi, Cosmo Pharmaceuticals NV) is an antiandrogenic topical therapy approved for acne vulgaris. It acts by competitively inhibiting dihydrotestosterone binding to cutaneous androgen receptors, thereby reducing sebum production and inflammation at the pilosebaceous unit.^1^ Although localized skin reactions have been reported in clinical trials,^2^ the development of perioral dermatitis with clascoterone use remains rarely described in the literature.

Conventional topical therapies for acne vulgaris—including retinoids, benzoyl peroxide, keratolytics, and topical antibiotics—target follicular hyperkeratinization and microbial colonization but do not address sebogenesis.^3^ Systemic treatments fall into 3 groups: antibiotics, antiandrogen therapies, and isotretinoin. Historically, only oral hormonal agents and isotretinoin have been shown to reduce sebum production.^4^ Clascoterone represents the first topical agent to target this androgen-mediated pathway in both male and female patients.

## Case report

A 25-year-old woman presented for management of her longstanding acne and hirsutism in the context of polycystic ovary syndrome. Her acne was previously well controlled with a combined oral contraceptive (COC); however, she transitioned to a levonorgestrel intrauterine device (Mirena) after commencing carbamazepine 800 mg daily for bipolar affective disorder due to its enzyme-inducing effect on hepatic cytochrome P-450, which reduces COC efficacy. Her only other medication was fluvoxamine 200 mg daily.

**Fig 1:**
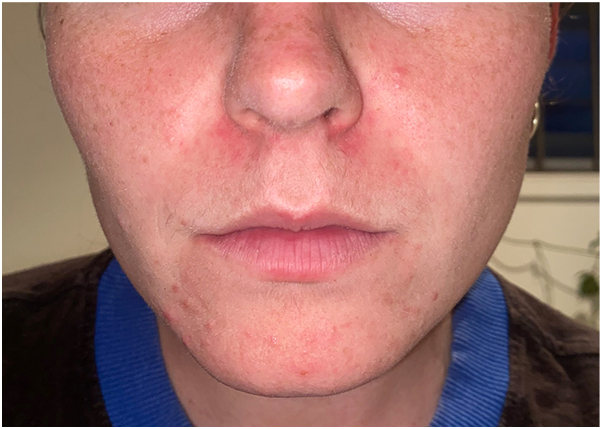
Erythematous papules confined to the perioral region, consistent with perioral dermatitis, developing after 7 weeks of topical clascoterone therapy.

She was commenced on spironolactone but, despite dosage escalation, demonstrated minimal clinical improvement. Isotretinoin was considered for 3 months but declined by the patient due to concerns about potential psychiatric side effects. At her 6-month review, topical clascoterone was introduced twice daily. After 7 weeks, she developed new-onset perioral dermatitis (Fig 1). Clascoterone was discontinued, and low-dose isotretinoin with topical tacrolimus was initiated, resulting in the resolution of her dermatitis.

## Discussion

Although effective, oral antiandrogen therapies are associated with numerous systemic adverse effects. The COC can be very effective in women with polycystic ovary syndrome; however, carbamazepine induces the hepatic cytochrome P-450 system, accelerating the metabolism of exogenous hormones. This interaction may cause breakthrough bleeding and reduce contraceptive efficacy by permitting ovulation.^5^ COC and spironolactone remain contraindicated in males due to feminizing effects.^2^ Isotretinoin, although highly efficacious, is a well-established teratogen,^4^ and its potential association with psychiatric disorders such as anxiety and depression remains controversial.^6^ Given the patient’s psychiatric history and personal preference, isotretinoin was initially avoided and later introduced cautiously at a low dose. Although antibiotics reduce follicular bacterial colonization and inflammation, they do not address the underlying pathogenesis of acne.

Clascoterone reduces sebum production and inflammation within the pilosebaceous unit through competitively inhibiting dihydrotestosterone binding to cutaneous androgen receptors.^1^ Its local metabolism contributes to a favorable safety profile, with minimal systemic absorption and fewer systemic adverse effects than oral antiandrogens.^1^ Clascoterone is rapidly hydrolyzed by skin and plasma esterases into cortexolone,^1^ which has weak glucocorticoid activity. This mild glucocorticoid effect may predispose to perioral dermatitis in susceptible skin. Alternatively, the vehicle formulation may be contributory, as it contains propylene glycol—a recognized sensitizer implicated in allergic contact dermatitis.^7^ Rare instances of irritant contact dermatitis have been reported in association with other excipients, including cetyl alcohol,^8^ citric acid, edetate disodium,^9^ and polysorbate 80.^10^

Perioral dermatitis has not previously been reported as an adverse effect of topical clascoterone. Although the agent is generally well-tolerated, clinicians should be aware of this potential complication to ensure prompt recognition and management, particularly when prescribing to patients with sensitive skin or a history of dermatitis.

## Conflicts of interest

None disclosed.
